# Exploring the Gain of Function Contribution of AKT to Mammary Tumorigenesis in Mouse Models

**DOI:** 10.1371/journal.pone.0009305

**Published:** 2010-02-19

**Authors:** Carmen Blanco-Aparicio, Marta Cañamero, Yolanda Cecilia, Belén Pequeño, Oliver Renner, Irene Ferrer, Amancio Carnero

**Affiliations:** 1 Experimental Therapeutics Programme, Spanish National Cancer Research Centre, Madrid, Spain; 2 Biotechnology Programme, Spanish National Cancer Research Centre, Madrid, Spain; Roswell Park Cancer Institute, United States of America

## Abstract

Elevated expression of AKT has been noted in a significant percentage of primary human breast cancers, mainly as a consequence of the PTEN/PI3K pathway deregulation. To investigate the mechanistic basis of the AKT gain of function-dependent mechanisms of breast tumorigenesis, we explored the phenotype induced by activated AKT transgenes in a quantitative manner. We generated several transgenic mice lines expressing different levels of constitutively active AKT in the mammary gland. We thoroughly analyzed the preneoplastic and neoplastic mammary lesions of these mice and correlated the process of tumorigenesis to AKT levels. Finally, we analyzed the impact that a possible senescent checkpoint might have in the tumor promotion inhibition observed, crossing these lines to mammary specific p53(R172H) mutant expression, and to p27 knock-out mice. We analyzed the benign, premalignant and malignant lesions extensively by pathology and at molecular level analysing the expression of proteins involved in the PI3K/AKT pathway and in cellular senescence. Our findings revealed an increased preneoplastic phenotype depending upon AKT signaling which was not altered by p27 or p53 loss. However, p53 inactivation by R172H point mutation combined with myrAKT transgenic expression significantly increased the percentage and size of mammary carcinoma observed, but was not sufficient to promote full penetrance of the tumorigenic phenotype. Molecular analysis suggest that tumors from double myrAKT;p53(R172H) mice result from acceleration of initiated p53(R172H) tumors and not from bypass of AKT-induced oncogenic senescence. Our work suggests that tumors are not the consequence of the bypass of senescence in MIN. We also show that AKT-induced oncogenic senescence is dependent of pRb but not of p53. Finally, our work also suggests that the cooperation observed between mutant p53 and activated AKT is due to AKT-induced acceleration of mutant p53-induced tumors. Finally, our work shows that levels of activated AKT are not essential in the induction of benign or premalignant tumors, or in the cooperation of AKT with other tumorigenic signal such as mutant p53, once AKT pathway is activated, the relative level of activity seems not to determine the phenotype.

## Introduction

The PTEN/PI3K pathway is one of the most altered in cancer. PTEN activity is lost by mutations, deletions or promoter methylation silencing at high frequency in many primary and metastatic human cancers [1], [2]. Germline mutations of PTEN are found in Cowden, Bannayan-Riley-Ruvalcaba and a Proteus –like syndromes, all of them familial cancer predisposition syndromes [3], [4], [5], [6]. The PI3KCA gene (coding for the p110α catalytic subunit of PI3K) have been described to be highly mutated in human tumors [2], [7]. The most frequently observed PI3-kinase mutations showed enhanced catalytic activity [8], able to constitutively activate AKT and produce transcriptional activation. It has also been reported an AKT1 somatic mutation in human breast, colorectal and ovarian cancers. This mutation activates AKT by means of pathological localization to the plasma membrane, stimulates downstream signaling, transforms cells and induces leukemia in mice [9]. Activation without mutations of PI3K and AKT are reported to occur in breast [10], [11], [12], ovarian [11], [13], [14], pancreatic [15], esophageal [16], thyroid cancer [17] and other cancers [2], [18].

Loss of PTEN function, as well as PI3K activation (either by growth factors or oncogenic mutations), results in accumulation of PIP3 triggering the activation of its downstream effectors, PDK1 and AKT. AKT activation stimulates cell cycle progression, survival, metabolism and migration through phosphorylation of many physiological substrates [19] [20], [21]. Deletion of AKT1 reversed the survival phenotype in PTEN null cells and abrogated its growth advantage [22]. Similarly, inactivation of AKT by dominant negative mutants inhibits the survival advantage provided by activated class I PI3K [23]. Disruption of AKT1 inhibits ErbB2-induced mammary tumorigenesis [24]. AKT1 deficiency delayed tumor growth and reduced metastasis. AKT1 null mammary epithelial tumor cells have also reduced proliferative capability with reduced cyclinD1 and increased p27 [24]. This suggests that AKT1 plays an important role in mammary tumorigenesis.

However, these contributions to tumorigenesis come from loss of function studies, corroborating that AKT activation is necessary for some tumorigenic signals. However, the situation is more complex when we try to reproduce tumorigenic phenotypes by gain of function of AKT signal.

Targeted deletion of PTEN in the mouse leads to constitutive activation of the PI3K/AKT pathway. Mice with a mammary-specific deletion of the PTEN gene displayed precocious lobulo-alveolar development, excessive ductal branching, delayed involution and severely reduced apoptosis [25]. PTEN null mammary epithelial cells were deregulated and hyperproliferative. Homozygous mutant females developed mammary tumors early in life. The constitutive activation of p110α in the epithelial cells of the mammary gland predisposes mammary glands to neoplastic transformation [26]. This mild tumor phenotype was enhanced when the mice were crossed with mice carrying an activating mutation of cdk4. Activation of AKT into mammary epithelial cells led to involution defects, however, non increased rate of tumors were observed despite the high expression levels of the constitutively active form of AKT [19], [27] [28].

In this regard, complete loss of PTEN in prostate also triggers cellular senescence which occurs through a p53-dependent mechanism [29]. Thus, complete loss of PTEN results in an indolent prostate cancer, due to the fact that PTEN inactivation in the prostate triggers a p53 cellular senescence response in vivo, thereby limiting the progression of cancer. Therefore, concomitant or sequential prostatic loss of PTEN and p53 results in a dramatic acceleration of prostate tumorigenesis. The rationale suggests that a p53-dependet senescent checkpoint is inhibiting malignant tumorigenesis in these models [30].

On the other hand, Majumder and colleagues, [31] recently published that a p27kip1-dependent checkpoint induces senescence upon AKT activation, and that the ablation of this checkpoint, allows the progression from PIN to carcinoma. Interestingly, p27kip1 (p27) upregulation in PIN lesions do not depend on AKT activation but was associated with alterations in cell polarity, architecture and adhesion molecules.

To investigate the mechanistic basis of the AKT-dependent mechanisms of breast tumorigenesis, we have explored the phenotype induced by activated AKT transgenes in a quantitative manner, we also explored the possible mechanism of senescence and the possibility that different target cells for oncogenic activation might be in the origin of AKT-dependent tumorigenesis. To that end we have generated several transgenic mice lines expressing different levels of constitutively active AKT in the mammary gland. We thoroughly analyzed the phenotype of these mice and correlated the process of tumorigenesis to AKT levels. We have analyzed AKT signalling induced phenotype in preneoplastic and neoplastic mammary lesions. Finally, to explore the possible mechanisms underlying the diverse phenotypes we analyzed the impact that a possible senescent checkpoint might have in the tumor promotion inhibition observed, we crossed these lines to p53(+/−) heterozygous mice, to mammary specific p53(R172H) mutant expression, and to p27 knock-out mice. Our findings revealed and increased preneoplastic phenotype depending upon AKT signaling which is not altered by p27 or p53 loss. However, p53 inactivation by R172H point mutation combined with myrAKT transgenic expression significantly increased the percentage and size of mammary carcinoma observed, however it is not sufficient to promote full penetrance of the tumorigenic phenotype. Molecular analysis suggests that tumors from double myrAKT;p53(R172H) mice proceed from acceleration of initiated p53(R172H) tumors and not from bypass of oncogenic-induced senescence induced by activated AKT.

## Results

### Transgenic myrAKT Lines

**Figure 1:**
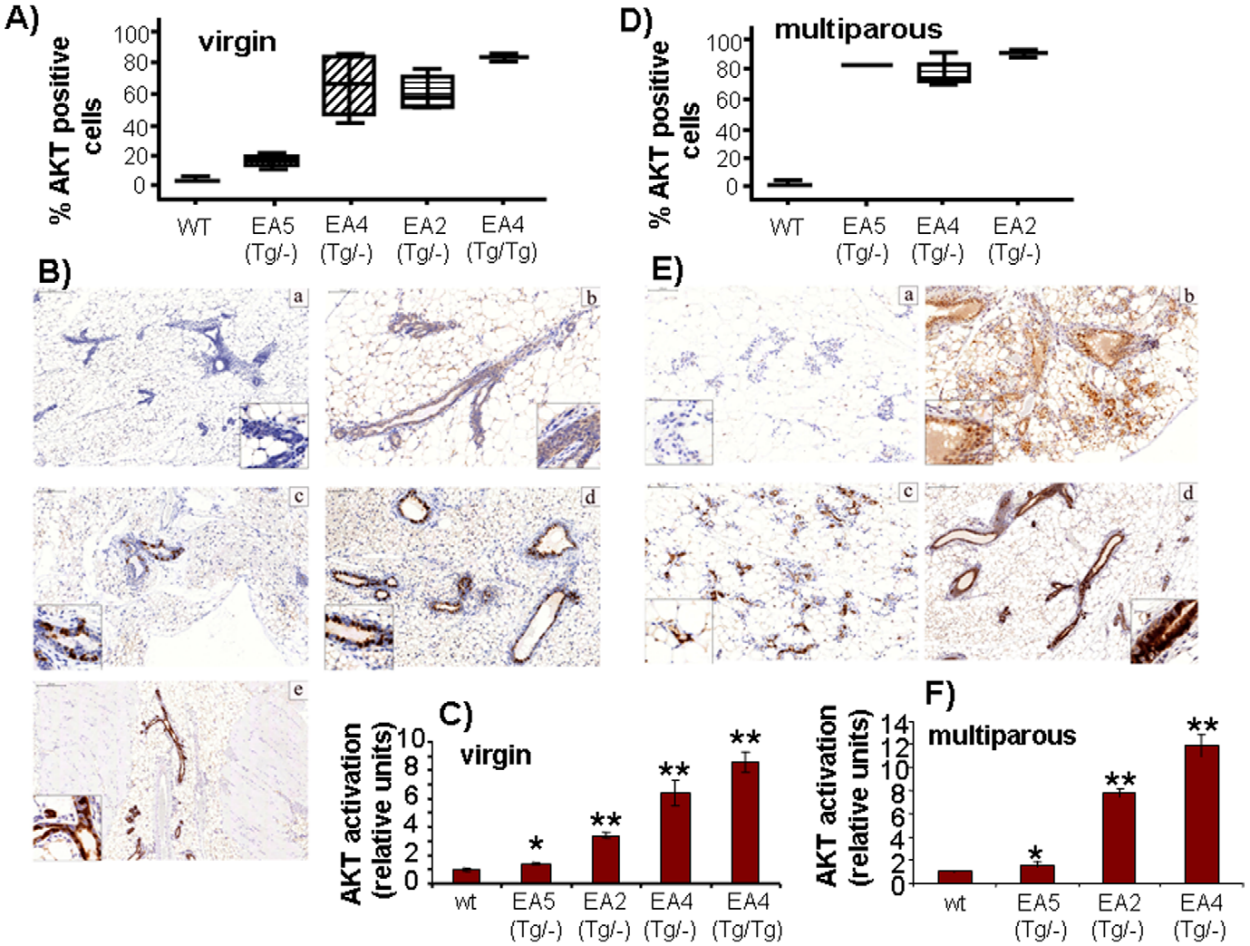
Expression of AKT-P S473 in the mammary gland of nulliparous and multiparous transgenic mice. (A) Percentage of ductal mamamry cells stained with AKT-P in heterozygous and homozygous nulliparous transgenic mice and wild-type littermates. (B) Immunohistochemical detection of phosphoS473 AKT1 in mammary glands of heterozygous and homozygous nulliparous transgenic mice and wild-type littermates. (C) Quantification of staining intensity of phosphoS473 AKT1 in mammary gland of heterozygous and homozygous nultiparous transgenic mice and wild-type littermates. (D) Percentage of ductal mamamry cells stained with AKT-P in heterozygous multiparous transgenic mice and wild-type littermates. (E) Immunohistochemical detection of phosphoS473 AKT1 in mammary glands of heterozygous multiparous transgenic mice and wild-type littermates. (F) Quantification of staining intensity of phosphoS473 AKT1 in mammary gland of heterozygous multiparous transgenic mice and wild-type littermates. The staining in epithelial cells was quantified using the NIH ImageJ software package. The difference between transgenic and wt is displayed as relative Intensity vs wt. P-value is 0.05 (unpaired Student's t test).

We generated several AKT-activated transgenic lines as previously described [27]. The different lines present variability in both, the levels of expression of activated AKT and the degree of chimaerism, number of cells per duct expressing the transgene. Out of 7 expressing transgenic lines we selected 3 of them, EA5, EA2 and EA4 that showed different levels of AKT activation (measured as AKT phosphorylation at S473) (Figure 1). We quantified the value of AKT activation and observed that EA5 line showed low AKT activation, just above the basal levels, however, EA2 and EA4 showed at least 5–7 fold activation. Due to chimaerism observed in most transgenic lines at difference from knock-ins models, the ducts also expressed variable percentage of cells expressing the transgene (Figure 1A and D). While EA5 showed low number of cells with the transgene, EA2 and EA4 presented an average of 60% cells per duct with AKT activated (Figure 1A). We did also generate the homozygous transgenic lines expressing two alleles of activated AKT. AKT levels increase in such homozygous lines (Fig 1C) although they do not double the levels of AKT activation. Similarly, homozygous lines increased the number of cells per duct expressing the transgene (Figure 1A). Since MMTV is activated by hormones it is known that multiparous animals increased the levels of expression of the transgenes driven by such promoter. Therefore, we maintained both one colony of virgin and another colony of multiparous females. As expected, multiparous females expressed higher levels of activated AKT (Figure 1F) and a percentage of cells above 90% expressing the transgene (Figure 1D). Therefore we can compare 12 different lines with different levels of AKT activation and a different percentage of cells per duct with transgene activation.

### Tumor Phenotype of Transgenic myrAKT Lines

The levels of AKT activation measured as S473 phosphorylation were functional in all lines, since mammary gland involution was delayed in all transgenic lines (Figure S1). However, despite genetic (one or two alleles) of physiological (virgin or multiparous) differences that contribute to different levels of AKT and chimaerism, no differences in the survival of transgenic lines were observed (Data not shown). We did not observe a statistical increase in the number of mammary tumors in any of the transgenic lines.

**Figure 2:**
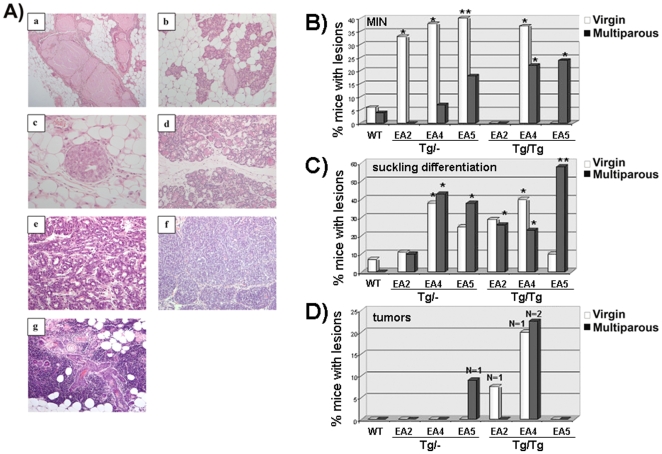
Incidence of preneoplastic and neoplastic mammary gland lesions of transgenic mice. A, H&E of mammary gland lesions of transgenic females expressing myrAKT: a) Cystic Dilatation/ectasia conteining proteinaceus fluid, b) Alveolar hyperplasia, c) Mammary intraepithelial neoplasia (MIN); d) Suckling differentiation; e and f) Carcinoma; e) Adenoaquamous tumor. B, Elevated incidence of MIN in heterozygous and homozygous virgin and multiparous females of lines EA2, EA4 and EA5. C,. Elevated incidence of suckling differentiation in heterozygous and homozygous virgin and multiparous females of lines EA2, EA4 and EA5. D, Elevated incidence of mammary tumors in heterozygous and homozygous virgin and multiparous females of lines EA2, EA4 and EA5. P-value is 0.05 (unpaired Student's t test).

An in deep analysis of the mammary glands of the animals indicated that the transgenic lines showed an increased percentage of intraductal mammary neoplasia (MIN) and suckling differentiation (Figure 2). This increase was observed in all transgenic lines without a clear correspondence with AKT levels or degree of chimaerism, indicating that high levels of AKT are not essential. Only a higher incidence of MIN in almost all transgenic lines is correlated with no pregnancies. A few animals with tumors were also observed, of variable type, with no statistical significance (Figure 2).

The molecular characterization of MIN indicated that MIN from wild type animals were different from to those arising in transgenic mammary glands. Transgenic MIN showed increased AKT phosphorylation and were positive for Progesterone receptor (PR), Estrogen Receptor (ER) and Estrogen Receptor phosphorylation (ER-P) (Figure S2).

The few tumors arising from transgenic animals were histologically heterogeneous but could be grouped in two main classes: adenosquamous (n = 1) and carcinomas of different types (n = 4) (Figure 2). Staining for molecular markers showed that carcinomas presented in general similar molecular pattern (Figure S2). All of them showed AKT phosphorylation and increased levels of ER, ER-P and PR. They also showed strong keratin 6 (CK6), E-cadherin and p63 staining.

Taken together our data indicated that increasing the levels of AKT in the mammary gland was not sufficient to produce a tumoral response in mice. Only a moderate increase in premalignant lesions was observed, and this was independent of the levels of AKT and the degree of chimaerism in the ductal epithelia.

### Senescence Markers

**Figure 3:**
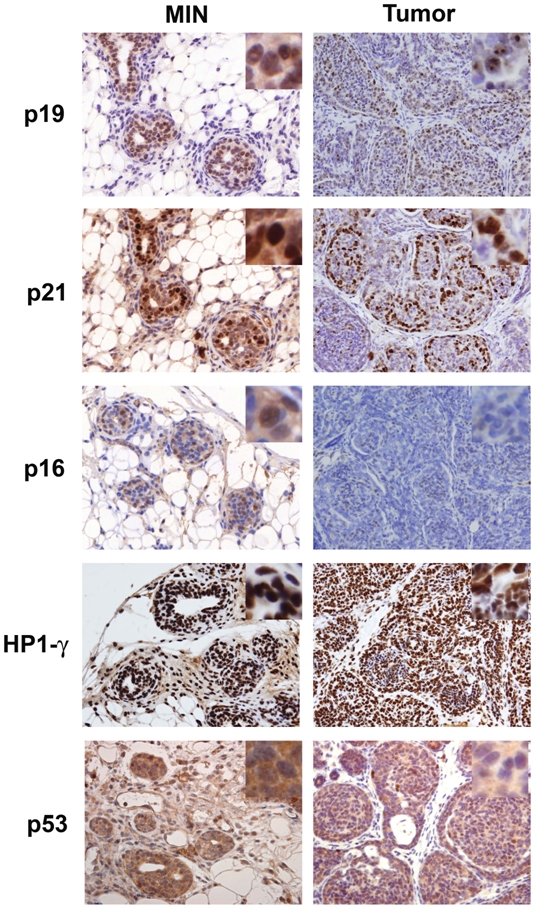
Expression of senescence markers in MIN and mammary tumors of myrAKT mice. Immunohistochemical staining of MIN and tumors with p19, p21, p16, Heterechromatin 1 gamma (HP1-γ) and p53. A 200× magnification was used and 400× magnification was inserted in the corner.

It has been proposed that the activation of AKT triggers a senescent checkpoint blocking the progression to full grown tumors [30], [32]. Therefore, we have explored whether premalignant lesions expresses senescent markers and whether these are lost in tumors (Figure 3). MIN from myrAKT transgenes clearly over expresses p19ARF, p21cip1 (here p21) and HP1γ markers of senescence. But these markers are maintained at similar levels in carcinomas (Figure 3). These markers are not expressed at similar levels in MIN from wild type animals suggesting specific oncogenic-induced senescent signals (Figure S3). On the other hand, p16INK4a (p16) is slightly induced in MIN from transgenic animal and lost in carcinomas (Figure 3) suggesting that Rb pathway may be a more important senescent checkpoint than p53 in AKT-derived tumors. To confirm this point we analyzed p53 stabilization as a correlate for p53 mutation by immunohistochemistry since it has been observed that most p53 mutations appear as strong nuclear p53 staining. Especially in p53 heterozygous mouse models, LOH generally occurs by p53 mutations. We observed that neither MIN nor macroscopic carcinomas showed p53 stabilization reflected by the low levels of nuclear p53 expression, correlating with wild type p53 (Figure 3). This result points to the existence of a tumor progression barrier different to p53.

### P27 Loss Does Not Contribute to AKT-Dependent Tumorigenesis

To explore whether a p27-dependent checkpoint induces senescence upon AKT activation, and that the ablation of this checkpoint, allows the progression from MIN to carcinoma, as has been suggested in prostate models [31], first, we checked whether p27 was activated in benign lesions and/or lost in tumors (adenosquamous or carcinoma) in our myrAKT mice. Immunohistochemistry staining showed that p27 was expressed in the normal mammary glands of wild type and AKT mice (Figure S4). The levels seemed increased in MIN from normal or myrAKT mice (Figure S4), although p27 levels were similar in both cases. However, these high p27 levels were maintained in carcinoma from myrAKT mice (Figure S4), indicating that p27 barrier was overcame during the process of tumorigenesis.

To complement the study whether p27 might play a role inhibiting the growth of AKT derived tumors we expressed myrAKT transgene into p27 hetorozygous and null backgrounds. MyrAKT;p27(−/−) mice develop pituitary tumors early in life and died without the possibility of studying the role of p27 absence in the mammary gland (Data not shown). Since p27 can also act as tumor suppressor in haploinsuficience we explored this possibility in our myrAKT mice. MyrAKT;p27(+/−) mice died slightly earlier than myrAKT mice, however, no mammary tumors were observed (Not shown). The analysis of the branching of mammary glands in these mice showed similar pattern than myrAKT, without further progression towards the tumoral phenotype (Figure S4). Our data suggest that although p27 might contribute to the AKT induced senescence it is not the primary determinant of arrest for these cells to stop growth in mammary gland.

However, given the correlative and not conclusive nature of these results, additional studies using tissue specific deletion of p27 in AKT-activated background are necessary to further strengthen this assertion.

### P53 Mutant Expression Cooperated with AKT Activation in the Generation of Breast Carcinoma

It has been proposed that AKT activation triggers senescence in primary cells and that this tumor suppressor mechanism might be dependent on the presence of p53 [29] [30]. However, our data from MIN and tumors of myrAKT models suggest that p53 pathway is not involved in the pre to malignant progression. However, other tumor models, such as head and neck, show p53 and AKT cooperation in tumorigenesis [33], [34].

**Figure 4:**
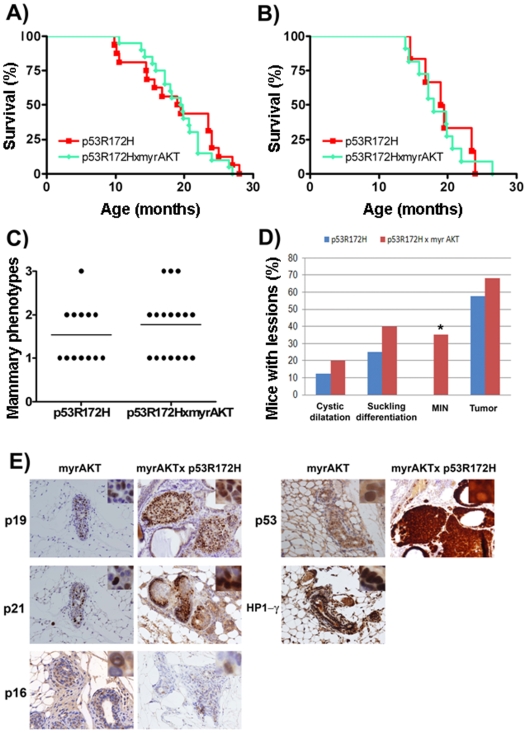
Survival and characterization of alteration in mammary gland of p53R172H transgenic mice. A) Tumor-free survival curves in p53R172H and p53R172H:myr-AKT transgenic mice. Survival curves were computed using the Kaplan-Meier product-limit method. The numbers of mice are the following: p53R172H transgenic mice(n = 16); p53R172H:myr-AKT transgenic mice (n = 18). B) Survival of mice that have developed mammary gland tumors. The numbers of mice are the following: p53R172H transgenic mice(n = 8); p53R172H:myr-AKT transgenic mice (n = 11). C) Mammary gland phenotypes of the p53R172H transgenic mouse lines observed after death. Whole mammary glands of dead or sacrificed (humane end point) mice were stained with carmine red to visualize the structure of the ducts and alveolae. Three different categories of phenotypes could be distinguished: (1) normal structure similar to the one of involuted multiparous female mice; (2) Cystic dilation (of the ducts) of more than 4 time; (3) increased number of ramifications and strongly increased alveolar size. Points, median of each mouse cohort. In all cases, mice with pituitary tumors were not considered. D) Incidence of preneoplastic and neoplastic lesions in p53R172H and p53R172H:myr-AKT transgenic mice. E) Immunohistochemical staining of MIN and tumors with p19, p21, p16, Heterechromatin 1 gamma (HP1-γ) and p53. A 200× magnification was used and 400× magnification was inserted in the corner.

To further explore whether this mechanism plays a role in our experimental system, we generated mice carrying the activated AKT transgene and the mutant p53(R172H) in the mammary ductal epithelia by crossing the MMTV-myrAKT with the wap-p53(R172H) line. Double heterozygous transgenes were analyzed in deep. Neither, total survival nor survival of mice with mammary tumors was altered in these mice (Figure 4). And the range of preneoplastic lesions did not vary in doubles myrAKT;p53(R172H) from p53 mutant mice alone (Figure 4). We observed that while p53(R172H) transgenic mice did not show MIN, doubles myrAKT;p53(R172H) showed 40% mice carrying MIN, percentage similar to the observed in virgin myrAKT transgenic lines (Figure 4). The transgenic lines with p53(R172H) are multiparous and the incidence of MIN in doble transgenic line is equivalent to the virgin myrAKT mice. Identical data was observed when myrAKT transgene was expressed in a p53(+/−) heterozygous background in virgin females (Figure S5). Therefore, p53 mutant does not alter the MIN to carcinoma transition in myrAKT1 transgenics. Moreover, multiparous females that expressed p53 mutant and myrAKT reached levels of MIN equivalent to virgin females.

To further explore the role of p53 in AKT-dependent MIN we compared the behavior of senescent markers p19ARF, p21 or p53 in MIN from myrAKT in wild type or p53(R172H) mutant backgrounds (Figure 4D), or in p53(+/−) heterozygous background (Figure S6). P19ARF is increased in all lines. However, it shows different levels among the different lines, being greatly overexpressed in the presence of mutant p53 as expected. p21 was equally increased in all lines while p53 showed clear staining in both lines expressing the mutant p53(R172H).

**Figure 5:**
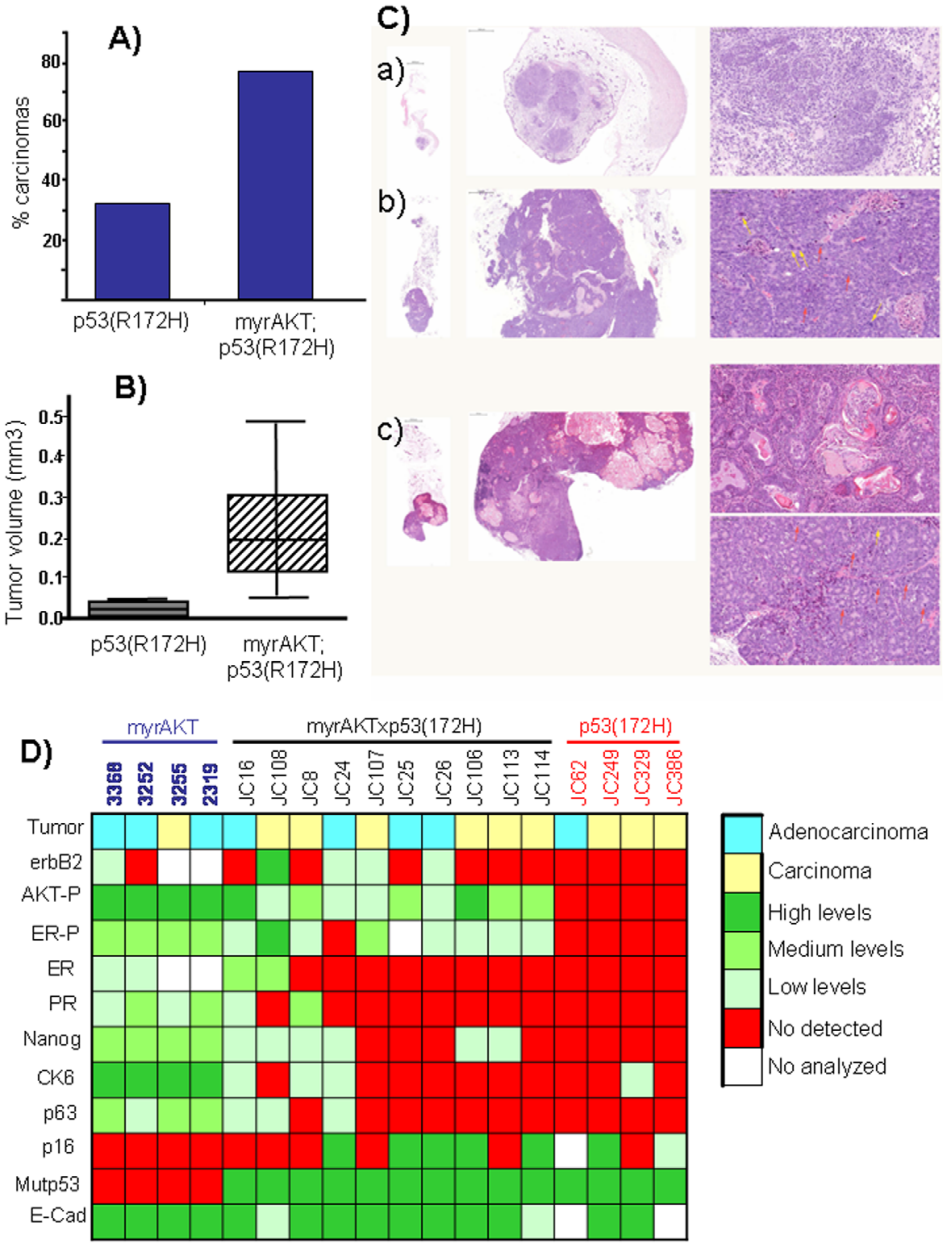
Incidence and characterization of mammary carcinoma in p53R172H and myrAKT;p53R172H transgenic mice. A) Incidence of carcinomas developed by transgenic mice. B) Tumor size of carcinomas developed by transgenic mice. C) Histological characterization of carcinomas. H&E staining of carcinomas from p53R172H (a) and myrAKT;p53R172H (b and c). Three different magnifications are shown for each tumor : 20×, 100× and 400× to compare the size of the tumors and the heterogeneity between then and in one tumor. a) adenocarcinoma, b) Solid carcinoma, c) Mixed carcinoma with acinar, glandular and squamous components. D) Heat map of the expression of several markers in carcinomas from myrAKT, p53R172H or doubles myrAKT;p53R172H.

Although we did observed a 30% p53(R172H) mice with carcinomas, the number of mice with carcinoma in doubles myrAKT;p53(R172H) increased to 80% (Figure 5A), tumors that showed bigger size. While in p53(R172H) mice carcinomas showed an average size of less than 0.03 cm^3^, in double myrAKT;p53(R172H) mice, carcinomas had an average volume of 0.2 cm^3^ (Figure 5B and C). Both however, showed clear p53 staining (confirming p53(R172H) mutant expression). Double myrAKT;p53(R172H) tumors also showed activated AKT, but in general the levels were lower than in myrAKT only tumors.

These data indicated that activated AKT cooperated with p53 mutant increasing the number and size of carcinomas but not the number of premalignant lesions, suggesting a more aggressive phenotype instead of a predisposition to develop a malignant lesion. However, the number of premalignant lesions, especially MIN, do not decrease as consequence of the transition to carcinoma, nor the double myrAKT;p53(R172H) transgenic mice showed full penetrance phenotype despite broad expression of transgenes in mammary ducts. Together with the data of no mutations in the p53, or p19ARF in tumors from myrAKT suggest that the constitutive activation of AKT induced a senescent barrier that is independent of p53. Therefore, the mutant p53-AKT observed cooperation might be due to an increase in proliferation induced by AKT activation in p53(R172H)-induced carcinomas.

To explore this possibility we compared the different tumors arising in the different cohorts. These tumors were analyzed immunohistologically for a set of common mammary tumor markers and evaluated by double blind observations by 2 independent pathologists. Discrete values between 0 (no expression) and 3 (higher levels) was assigned to each tumor and the correlation between different tumors analyzed by using the function hcluster (package amap) of the free statistical software R. The different tumors analyzed showed very high pathological variability across all transgenic lines. Carcinomas and adenocarcinomas with different degrees of mixed, glandular, acinar or solid pathological features were observed in all lines. No specific morphological pattern was assigned to any genetic background (Figure 5D). We observed that while AKT levels were very high (3) in all myrAKT tumors, tumors from double myrAKT;p53(R172H) transgenic mice were more heterogeneous but in general showed lower levels of activated AKT (Figure 5D, Figure S7). Furthermore, ER phosphorylation but not ER expression was associated with AKT activation in agreement with previous results indicating the relevance of ER phosphorylation by AKT [35] [36] [27] [26]. All tumors showed clear E-cadherin staining as reflection of their epithelial origin. CK6, nanog, p63, ER y PR are expressed in all myrAKT tumors but only in a very small (aprox 20%) tumors from double myrAKT;p53(R172H), and mostly low levels (Figure S7). However it absence in most double myrAKT;p53(R172H) tumors correlated with the observed in p53(R172H) induced carcinomas (Figure 5D, Figure S7). Furthermore, p63, CK6 and nanog levels are homogeneous and higher in myrAKT tumors while tumors with mutant p53 (from p53(R172H) and myrAKT;p53(R172H) mouse lines) are heterogeneous and show lower levels of expression of these proteins.

ErbB2 (Her2, neu) overexpression is observed in 20–30% human breast cancer [37]. In mouse models loss of PTEN function lead to elevated erbB2 protein levels [38]. We have tested whether in our model AKT activated transgene induced erbB2 protein. Our data showed a few ErbB2 barely positive tumors which correlated with no expression of ER and PR and high hystopathological grade. Only one tumor, probably outlayer, showed high ErbB2 positivity.

The overall picture shows that tumors from double myrAKT;p53(R172H) transgenic mice are more close to tumors from p53(R172H), while tumors from myrAKT are different. However, the number of tumors is very small and a much higher number of tumors are necessary to analyze to further support our conclusions.

Taken together, our experiments suggest that although tumor proliferation may be blocked by the presence of wild type p53, p53-dependent senescence might not be the only tumor suppressor mechanism impeding tumorigenesis in the mammary glands of myrAKT transgenic mice. The cooperation observed between activated AKT and mutant p53 seems to indicate that AKT enhances the proliferation of mutant p53-induced tumors.

## Discussion

Elevated expression of AKT has been noted in a significant percentage of primary human breast cancers, mainly as a consequence of PTEN/PI3K pathway deregulation. The PTEN/PI3K pathway is probably the most frequently mutated pathway in human cancer. Furthermore, tyrosine kinase membrane receptors are also mutated or activated (ie: ErbB2, EGFR) contributing to AKT deregulation in mammary tumors. Different works analyze the necessity of the AKT signal in mammary tumorigenesis induced by different oncogenic signals (reviewed in [39] [40] [41]). However, the enforced AKT activation found in most mammary tumors suggest that there is also a sufficient signal to promote tumorigenesis. However, this point is still controversial since mammary models do not completely recapitulate the expected tumorigenic phenotype [39].

Several hypotheses, however, have been proposed to explain this apparent phenomenological discrepancy. Transgenic expression of activated AKT does not reach appropriate levels to induce tumorigenesis. Either they are too low to activate oncogenic pathway or too high activating senescence. Enforced AKT activation leads to p53-dependent senescence, like for PTEN loss [29], or p27-dependent senescence [31]. Finally, It is also possible that transgenic AKT activation does not occurs in the appropriated target cell (ie: it is possible that MMTV promoter used in most of the transgenic mouse models it is not activated in the cells susceptible to generate a tumor).

To investigate the mechanistic basis of the AKT-dependent mechanisms of breast tumorigenesis, we have generated several transgenic mice lines expressing different levels of constitutively active AKT in the mammary gland. Mice expressing activated AKT showed an increased percentage of suckling differentiation even in nulliparous females. In humans high parity has generally been associated with low breast cancer risk in several epidemiological studies [42] [43] [44]. In mice ablation of AKT1 dramatically delays mammary gland differentiation during pregnancy and lactation and promotes apoptosis and accelerates involution what correlates with defects in Stat5 phosphorylation [45]. Maybe the expression of an activated AKT promotes a differentiation of the mammary gland that protects from tumor development.

Moreover, we thoroughly analyzed the phenotype of these mice and correlated to the process of tumorigenesis by either preneoplastic or neoplastic lesions. AKT activation increased the number of benign lesions but neither AKT activated levels or number of cells with AKT activation in the mammary duct correlated with lesion number. Only myrAKT multiparous females from almost all transgenic lines showed an incidence of benign lesions, especially premalignant MIN, lower than nulliparous females. These data suggest that the absence of tumors in AKT-activated mice is not a consequence of inappropriate AKT levels in the transgene. We can also exclude that the AKT is not activated in the right target cells (ie: cancer prone cell) since we can increase the number of cells per duct with AKT activation in different lines but we can not observe a parallel increase in benign or malignant lesions. Therefore, our results suggest the existence of a tumor suppressor barrier that prevents tumorigenesis once AKT is activated. Clearly, p53 and p27 dependent senescence are mayor mechanisms proposed to achieve this goal.

To explore these mechanisms we have analyzed different molecular markers of senescence in the benign and malignant lesions formed in AKT-activated mice. We have found that p19ARF, p21, p27, p16 and HP1γ seems to be activated in MIN but only p16 disappears in tumors, indicating that pRb might constitute the senescent checkpoint which needs to be overcame in these AKT-dependent lesions to produce tumors. Interestingly, half of tumors containing mutant p53 also lost p16.

To further explore the relevance of p27 and p53 checkpoints, we generated transgenes with activated AKT in p27(+/−), p53 (+/−) or p53(R172H) mutant backgrounds. Our findings revealed that the enhanced AKT-dependent preneoplastic phenotype was not altered by p27 reduction or p53 activity loss. However, p53 inactivation by R172H point mutation combined with myrAKT transgenic expression significantly increased the percentage and size of mammary carcinomas. However it was not sufficient to promote full penetrance of the tumorigenic phenotype.

These data indicated that activated AKT cooperated with p53 mutant increasing the number and size of carcinomas but not the number of premalignant lesions. However, the number of premalignant lesions, MIN, do not decrease as consequence of the transition to carcinoma, nor the double myrAKT;p53(R172H) transgenic mice showed full penetrance phenotype despite broad expression of transgenes in mammary ducts. Moreover, AKT activation in parous females decrease the incidence of MIN, but in the presence of p53 mutant this protection was not observed. Together with the data of no mutations in the p53 nor p19ARF in tumors from myrAKT suggest that the constitutive activation of AKT induced a senescent barrier that is independent of p53. Therefore, the mutant p53-AKT observed cooperation might be due to an increase in proliferation induced by AKT activation in p53(R172H)-induced carcinomas. The molecular comparison of tumors from myrAKT, p53(R172H) or double myrAKT;p53(R172H) mice suggests that myrAKT;p53(R172H) tumors seem to proceed from p53(R172H) expressing cells and that AKT accelerated the process of tumorigenesis, rather than from cells with activated AKT where the p53 brake has been eliminated.

On the other hand, R172H p53 mutant, equivalent to human R175H, is a structure defective mutant whose point mutation determines an important conformational alteration [46]. The presence of conformational mutants has been strongly associated with breast cancer patients [47] [48] [49]. Mutant p53 proteins do not represent the mere loss of wild type p53 activity, but gain new additional oncogenic functions which contribute to the development, maintenance and spreading of cancer [46] [50], [51] [52]. It has been shown that mutant p53 triggers different pathways that represent the molecular basis of the gain of function activity [52]. These scenarios might favor the increase of MIN detected in multiparous double transgenic mice that do no progress to carcinoma due to the senescence induced by AKT independent of p53. On the other hand, the expression of mutant p53 might contribute to tumor initiation, while active AKT background might contribute to tumor progression through several of the pleiotropic effects induced by AKT activity (Reviewed in [53], [54], contributing to the large increase in size observed in carcinomas. On the other hand, increased proliferation can also contribute to the increased number of carcinomas by increasing carcinoma detection.

Taken together, our experiments suggest that although tumor proliferation may be blocked by the presence of wild type p53, p53-dependent senescence might not be the only tumor suppressor mechanism impeding tumorigenesis in the mammary glands of myrAKT transgenic mice. This is confirmed by the lost of p16 in half of tumors with mutated p53.

These data fully agree with our hypothesis that pRb might constitute the main senescent checkpoint which needs to be overcome in these AKT-dependent lesions to produce tumors. This hypothesis also correlated with the works of Yu et al [55], Landis et al [56] and Reddy et al [57] reporting the necessity of CDK4 activity for mammary tumorigenesis. Our data point towards loss of CDK4 inhibitor p16 is the genetic alteration in the hyperplasia to tumor transition in the AKT activated transgene. In fact, previous results from our group showed that activated p110α transgenic expression in CDK4(R24C) mutant background produces an increase in preneoplastic lesions and in tumor formation [26].

In human breast tumors, activation of cyclin D1 is a common event. Amplification of the gene has been detected in about 15% of breast cancers, while overexpression of cyclin D1 at mRNA and protein levels is seen in up to 50% of primary tumors, mostly ER-positive and well-differentiated tumors [58] [59]. Furthermore, joint overexpression of cyclin D and PI3K pathway activation, either directly or indirectly through membrane receptors, is a common event described in human tumors [60] [40], [61].

Since breast cancer is notable for the high incidence of cyclin D1 aberrations with up to 50% of tumors expressing elevated levels of the protein (reviewed in [59] [59] [62]), we can expect to find p16 defects among the remaining tumors. To date, there have been reports suggesting that p16 may be affected by methylation in primary breast cancers with quoted frequencies of 15–30% [63] [64] [65] [66] [67] and there is a relatively high rate of LOH at 9p21 [68] [69] [63]. However, due the complex nature of the 9p21 loci containing also the p14ARF and p15INK4b tumor supressors, it is difficult to score the true relevance of p16 in this loci loss. Furthermore, a high number of breast cell lines show abnormalities, including the spontaneously immortalized line MCF10A [65] [70]. In HMEC, p16 silencing by promoter methylation has been reported as one of the initial events occurring to allow cell proliferation, contributing to alleviate senescent checkpoint [71].

In our model, cyclin D1amplification or p16 lost in breast tumors could be equivalent in maintaining CDK4 activity and therefore inactivating pRb checkpoint.

Taken together, our experiments suggest that although tumor proliferation may be blocked by the presence of wild type p53, p53-dependent senescence might not be the only tumor suppressor mechanism impeding tumorigenesis in the mammary glands of myrAKT transgenic mice. It seems that in this model, pRb pathway is more important checkpoint controlling senescence. The cooperation observed between activated AKT and mutant p53 seems to indicate that AKT enhances proliferation of mutant p53-induced tumors

## Materials and Methods

### Ethics Statement

All animal experiments were done under the experimental protocol approved by the Institutional Committee for Care and Use of Animals of the Spanish National Cancer Research Centre which complies with European legislation on the care and use of animals, NIH guidelines for the use of laboratory animals, and related codes of ethic practice. The ethics committee for care of animals specifically approved this study.

### Generation and Analysis of Transgenic Mice

Transgene construction is described in Blanco-Aparicio et al, 2007a. Transgenic mice were generated by the microinjection of the 4.7 kb fragment from the digested pMMTV-myrAKT1 with AatII+SalI, (this fragment contains the MMTV promoter, myrAKT1 sequences and SV40 poly-adenylation signal) into the pronucleus of single-cell embryos isolated from super-ovulated B6/CBA mice, according to standard procedures by the Transgenic Unit, CNIO. Embryos that survived the microinjection were implanted into pseudo-pregnant females and allowed to develop to term. All the crosses where kept in C57BL6 genetic background. Genotyping of the transgenic MMTV-myrAKT1 mice was performed by PCR of tail DNA. For details, see Blanco-Aparicio et al, 2006. p53 KO mice were obtained from Jackson laboratories. p27 were a kind gift of Dr. Manuel Serrano and p53mutant R172H were obtain from mouse models of human cancers consortium mouse repository.

### Whole Mounts

Sacrified females (human end point) and 3 days after weaning pregnant females were euthanized using CO_2_, and mammary glands were harvested, placed on dry, silanized glass slides and fixed overnight in 1∶3∶6 parts of glacial acetic acid∶chloroform∶100% ethanol. Tissues were rehydrated through successive incubations with 70% ethanol followed by distilled water, and stained with Carmine red alum overnight. Tissues were then dehydrated through successive incubations in graded ethanol followed by mixed xylenes and mounted in Permount® (Fisher Scientific, Puerto Rico).

### Histopathology and Immunohistochemistry

Mammary glands were fixed in 10% buffered formalin and embedded in paraffin. For histopathologic analysis, they were sectioned (2.5 µm) and stained with H&E. Slideswere baked overnight at 55°C, deparaffinized in xylene, rehydrated, and washed with PBS. Epitope retrieval was performed in sodium citrate (pH6.1). Endogenous peroxide activity was quenched with 1.5% hydrogen peroxide in methanol for 10 min and incubation (40 min) with the primary antibodies: anti-phosphoS473AKT1 (Epitomics) diluted 1∶175; anti-cytokeratin 6 (Covance) diluted 1∶1000; anti p63 (Thermo Scientific) 1∶100; anti-nanog (Novus Biologicals) 1∶50; anti-p27 (Thermo Scientific) diluted 1∶125; anti-p19 (St Cruz Biotechnoly) 1∶50; anti-p21 (Santa Cruz Biotechnology) diluted 1∶350;anti erbB2 (Spring Bioscience) diluted 1∶15, rabbit polyclonal anti-p53 (Novocastra, Newcastle, UK) diluted 1∶200; anti phosphoS167ER*_α_* (New England Biolab) diluted 1∶25; anti ER alpha (Master Diagnostica) prediluted; anti progesterone receptor (Thermo Scientific) prediluted, anti-E-cadherin (BD Transduction Laboratories,) diluted 1∶150;antip16 (St. Crz Biotechnoogy) diluted 1∶75; horseradish peroxidase goat anti-rabbit IgG (Dako) diluted 1∶50; horseradish peroxidase goat anti-mouse IgG (Jackson Immunoresearch, West Grove, PA) diluted 1∶50; horseradish peroxidase rabbit anti-goat IgG (Dako) diluted 1∶50. After incubation, immunodetection was carried out with the peroxidase-based PAP system (DAKO) using diaminobenzidine as substrate. Incubations omitting the specific antibody were used as a control of the technique.

### Western Blot Analysis

Total protein was extracted from snap-frozen tissues by homogenization using a Dounce homogeneizer in 1 ml extraction buffer (25 mM Tris, pH 7.5, 150 mM NaCl, 1% TritonX-100, 15 mM EDTA, 10 mM NaF, 2 mM TAME, 5 mM benzamide, 10 µg/ml aprotinin, 40 µg/ml bestatin, 10 µg/ml EA4, 300 µg/ml phosphoramidon, 50 µg/ml antipain, 2.5 mM Na4P2O7, 15 mM pNPP, 1 mM DTT, 60 mM ß-glycerophosphate, 10 µg/ml leupeptine, 0.14 µg/ml chymostatin, 0.7 µg/ml pepstatin, 1 mg/ml pefabloc and complete protease inhibitor cocktail tablet (Roche molecular Biochemicals, Spain). Homogenates were centrifuged at 10 000 g for 10 min at 4°C twice, and fat was removed from the surface. Final supernatants were analyzed by western blot as described previously (28).

### Statistical Analysis

All statistics were analyzed using the SPSS statistical package (version 13.0 for Windows). Statistical analysis of tumor–free survival curves included calculation of Kaplan–Meier distributions of survival of two different groups of mice and comparison by a two-sided log-rang test. T de Student tests were conducted to test the different levels of expression of activated myrAKT, to test the different incidence of MIN, suckling differentiation and tumors in all the transgenic line vs wt mice. Spearman rank tests were conducted to test the differential incidence of epithelial and non-epithelial tumors between the different groups of treated mice. A P-value of <0.05 was considered statistically significant.

Hierarchical clustering was performed using the function hcluster (package amap) of the free statistical software R [72], as previously reported in [73]

## Supporting Information

Figure S1 Mammary gland involution is altered in transgenic females. The regression of the mammary glands of transgenic and wild-type littermates was determined 3 d after weaning of the first litter. Of each mouse, a whole mount preparation, followed by red carmine staining, was performed using the right hind leg mammary gland, whereas sections of the left hind leg mammary gland were stained with H&E or with the antibody against phosphorylated AKT. For all three transgenic mouse lines, a reduced involution of the mammary glands could be detected, which correlated with an elevated level of AKT phosphorylation.
(0.95 MB PPT)

Figure S2 Molecular characterization of MIN and mammary tumors in wt and myrAKT mice. Immunohistochemical staining of MIN and tumors with AKT Ser473, cytokerin 6, p63, progesterone receptor (PR), estrogen receptor alpha (ER) and phosphoS167ER -. A 200× magnification was used and 400× magnification was inserted in the corner.
(2.70 MB PPT)

Figure S3 Expression of senescence markers in MIN of wt and myrAKT mice. Immunohistochemical staining of MIN with p19, p21. A 200× magnification was used and 400× magnification was inserted in the corner.
(0.41 MB PPT)

Figure S4 p27Kip1 is expressed in mammary gland lesions of myrAKT mice and its lost does not promotes tumorigenesis. A) Immunohistochemical staining with p27 of wt normal mammary gland (a)and wt MIN (c)and myrAKT normal mammary gland (b), myrAKT Min (d), myrAKT tumors (e,f). A 200× magnification was used and 400× magnification was inserted in the corner B) Tumor-free survival curves in p27KO (+/−) and p27KO (+/−):myr-AKT transgenic mice. Survival curves were computed using the Kaplan-Meier product-limit method. The numbers of mice are the following: p27KO (+/): transgenic mice(n = 7); p27KO (+/−):myr-AKT transgenic mice (n = 12). C) Mammary gland phenotypes of the myr AKT and p27KO (+/−);myrAKT transgenic mouse lines observed after death. Whole mammary glands of sacrificed (humane end point) mice were stained with carmine red to visualize the structure of the ducts and alveolae. Four different categories of phenotypes could be distinguished: (1) normal structure similar to the one of young virgin female mice; (2) Cystic dilation (of the ducts) of more than 4 time; (3) increased number of ramifications and strongly increased alveolar size (4) Extreme alveolar hyperproliferation covering the space between the ducts to more than 70%. Points, median of each mouse branching observed.
(1.76 MB PPT)

Figure S5 Survival and characterization of alteration in mammary gland of myrAKT transgenic mice in a heterozygous p53 (+/−) background. A) Tumor-free survival curves of p53(+/−), myrAKT and the double transgenic p53(+/−);myrAKT transgenic mice. Survival curves were computed using the Kaplan-Meier product-limit method. The numbers of mice are the following: p53(+/−) (n = 32), p53(+/−);myr-AKT(n = 49) and myrAKT (n = 12). B) Survival of mice that have developed mammary gland tumors. The numbers of mice are the following: p53(+/−)(n = 3); p53(+/−);myrAKT (n = 3). C) Incidence of preneoplastic and neoplastic lesions in p53(+/−) and p53(+/−);myr-AKT transgenic mice.
(0.61 MB PPT)

Figure S6 Expression of senescence markers in MIN from myrAKT in wild type, p53(+/−) or p53R172H background. Immunohistochemical staining of MIN with p19, p21 and p53. A 200× magnification was used and 400× magnification was inserted in the corner.
(0.74 MB PPT)

Figure S7 Molecular characterization of carcinomas in myrAKT, p53R172H and myrAKT;p53R172H transgenic mice. Immunohistochemical staining with AKT Ser473, cytokerin 6, p63, progesterone receptor (PR), estrogen receptor alpha (ER), phosphoS167ER, p53, nanog and E-Cadherin-. A 200× magnification was used and 400× magnification was inserted in the corner.
(8.57 MB PPT)
