# Supplementary figures and images for: Exploring the Gain of Function Contribution of AKT to Mammary Tumorigenesis in Mouse Models
Source: PLoS One. 2010 Feb 19;5(2):e9305. doi: 10.1371/journal.pone.0009305 (PMC2824815; doi:10.1371/journal.pone.0009305)

## Slide 1
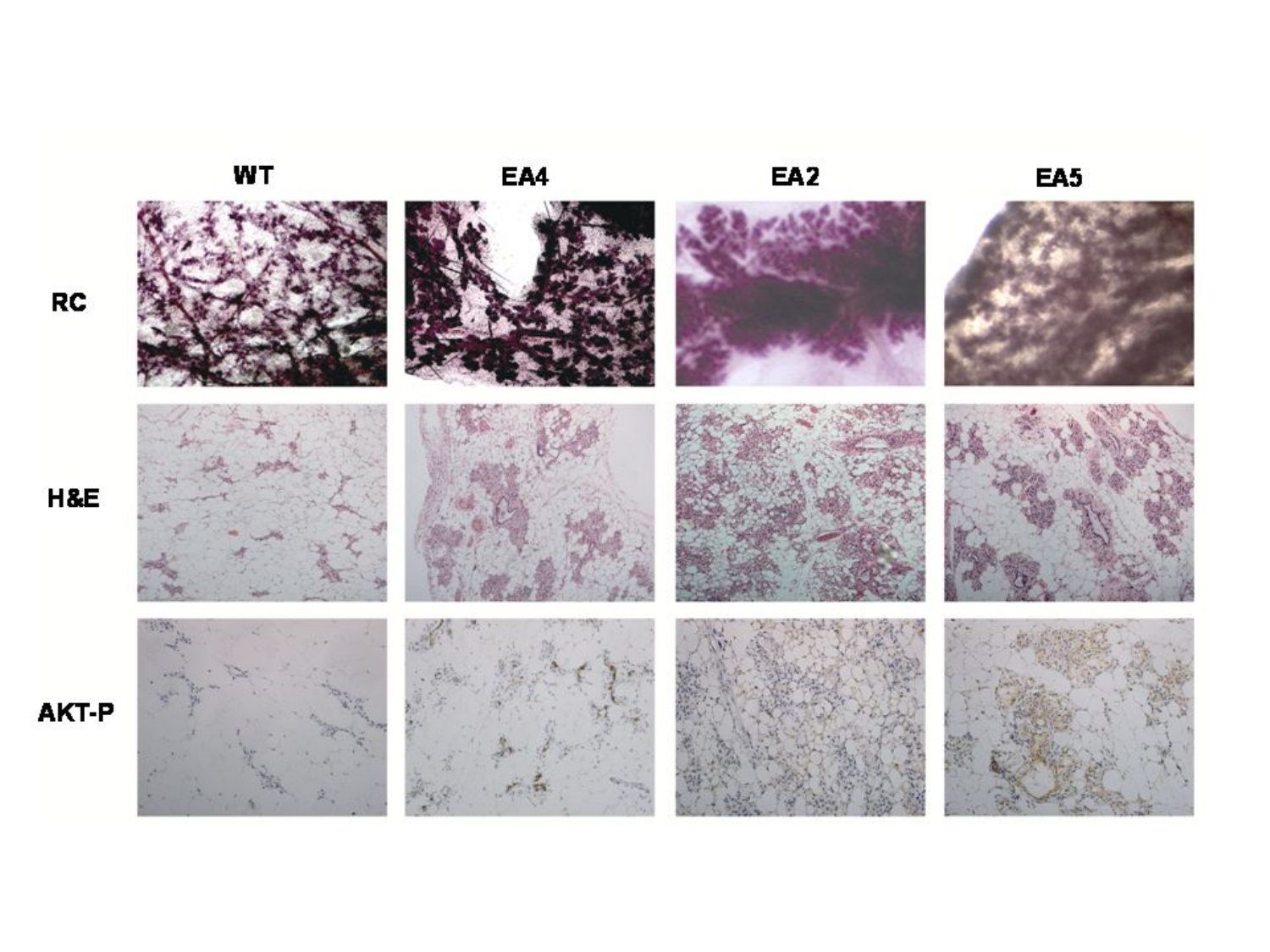

#

Supplement: Figure S1 — Mammary gland involution is altered in transgenic females. The regression of the mammary glands of transgenic and wild-type littermates was determined 3 d after weaning of the first litter. Of each mouse, a whole mount preparation, followed by red carmine staining, was performed using the right hind leg mammary gland, whereas sections of the left hind leg mammary gland were stained with H&E or with the antibody against phosphorylated AKT. For all three transgenic mouse lines, a reduced involution of the mammary glands could be detected, which correlated with an elevated level of AKT phosphorylation. (0.95 MB PPT) [file pone.0009305.s001.ppt]

## Slide 1
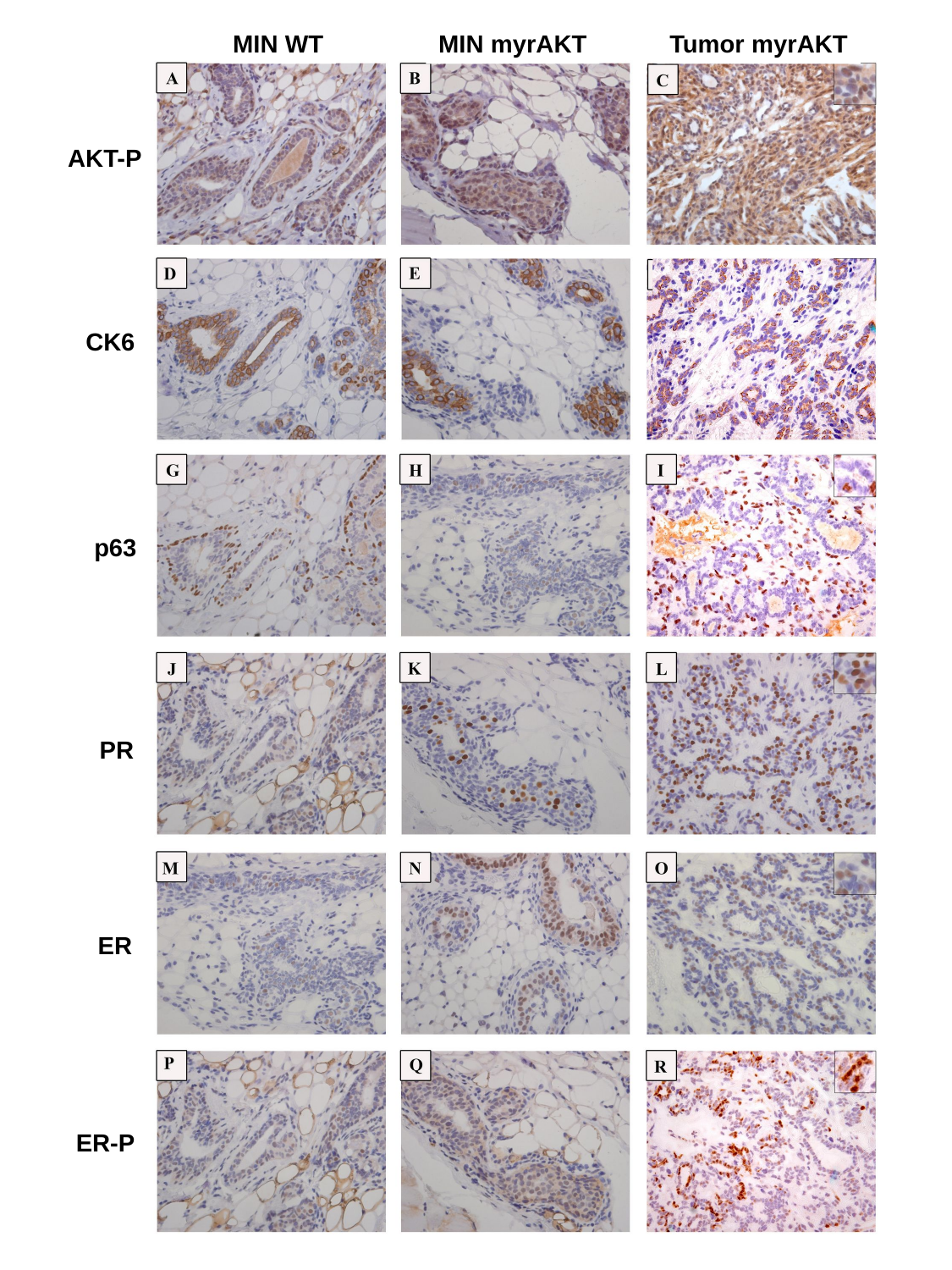

MIN WT
MIN myrAKT
Tumor myrAKT
AKT-P
CK6
p63
PR
ER
ER-P

Supplement: Figure S2 — Molecular characterization of MIN and mammary tumors in wt and myrAKT mice. Immunohistochemical staining of MIN and tumors with AKT Ser473, cytokerin 6, p63, progesterone receptor (PR), estrogen receptor alpha (ER) and phosphoS167ER -. A 200× magnification was used and 400× magnification was inserted in the corner. (2.70 MB PPT) [file pone.0009305.s002.ppt]

## Slide 1
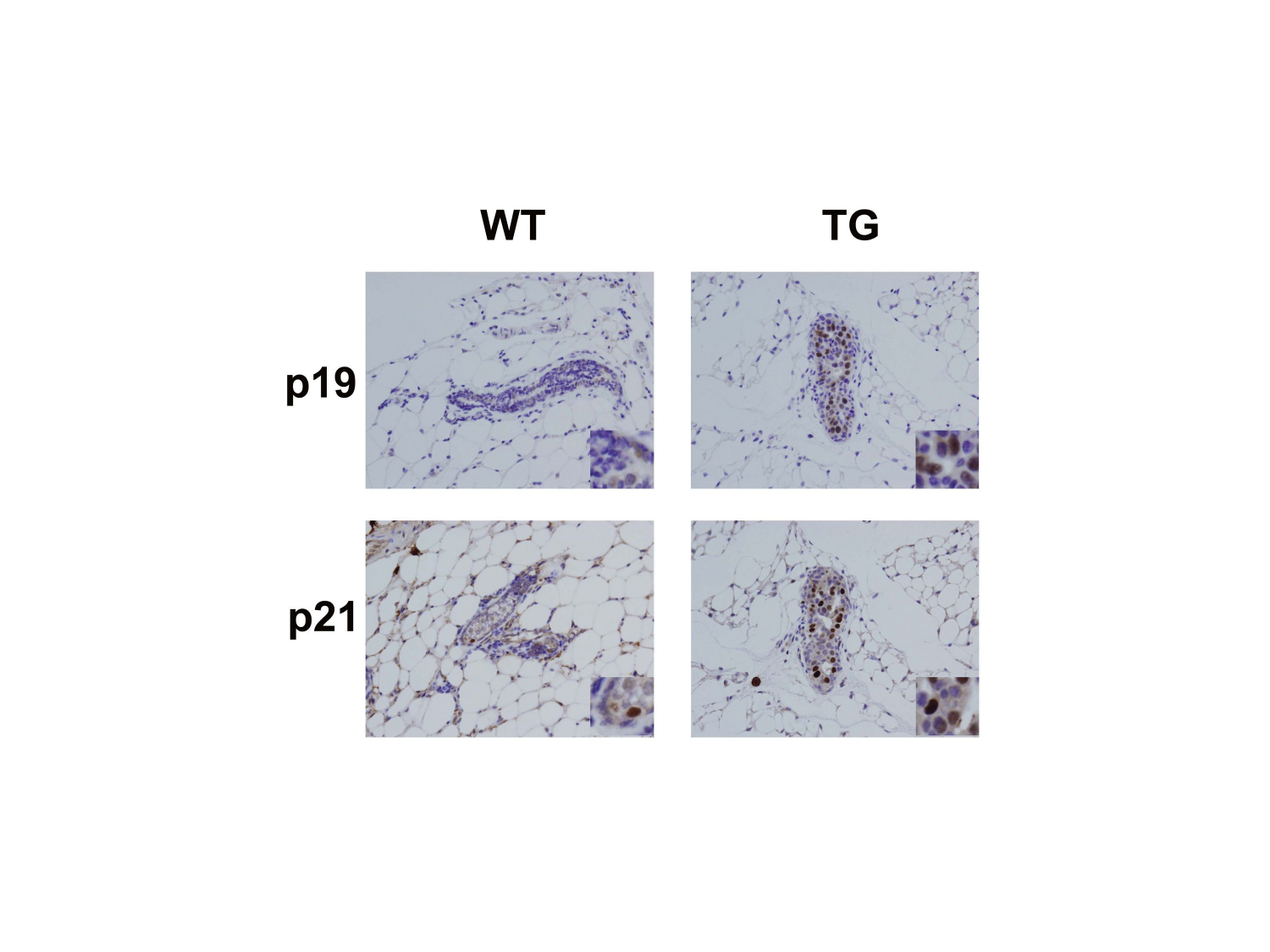

Supplement: Figure S3 — Expression of senescence markers in MIN of wt and myrAKT mice. Immunohistochemical staining of MIN with p19, p21. A 200× magnification was used and 400× magnification was inserted in the corner. (0.41 MB PPT) [file pone.0009305.s003.ppt]

## Slide 1
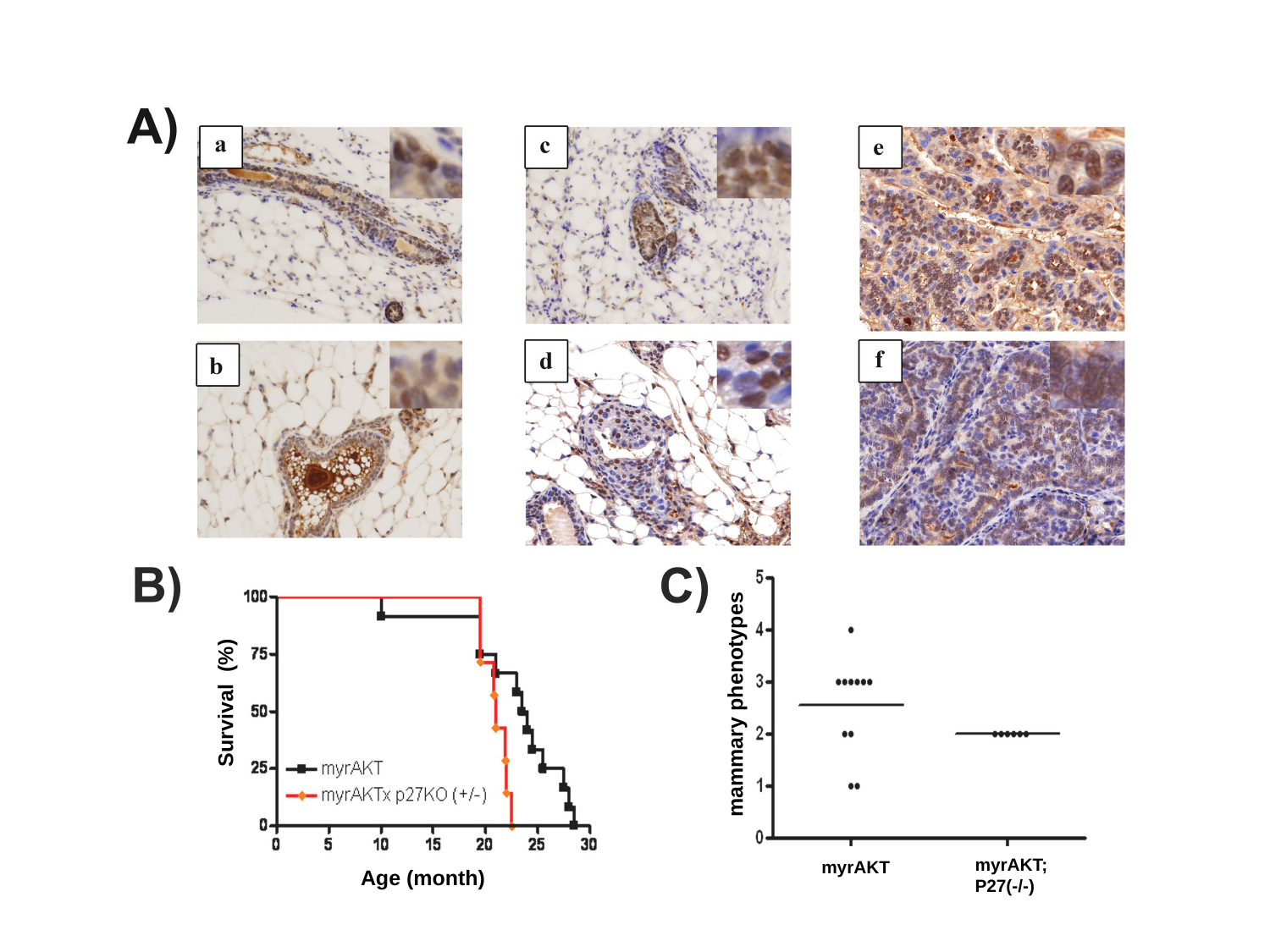

Survival (%)
 mammary phenotypes
myrAKT;
P27(-/-)
myrAKT
 Age (month)

Supplement: Figure S4 — p27Kip1 is expressed in mammary gland lesions of myrAKT mice and its lost does not promotes tumorigenesis. A) Immunohistochemical staining with p27 of wt normal mammary gland (a)and wt MIN (c)and myrAKT normal mammary gland (b), myrAKT Min (d), myrAKT tumors (e,f). A 200× magnification was used and 400× magnification was inserted in the corner B) Tumor-free survival curves in p27KO (+/−) and p27KO (+/−):myr-AKT transgenic mice. Survival curves were computed using the Kaplan-Meier product-limit method. The numbers of mice are the following: p27KO (+/): transgenic mice(n = 7); p27KO (+/−):myr-AKT transgenic mice (n = 12). C) Mammary gland phenotypes of the myr AKT and p27KO (+/−);myrAKT transgenic mouse lines observed after death. Whole mammary glands of sacrificed (humane end point) mice were stained with carmine red to visualize the structure of the ducts and alveolae. Four different categories of phenotypes could be distinguished: (1) normal structure similar to the one of young virgin female mice; (2) Cystic dilation (of the ducts) of more than 4 time; (3) increased number of ramifications and strongly increased alveolar size (4) Extreme alveolar hyperproliferation covering the space between the ducts to more than 70%. Points, median of each mouse branching observed. (1.76 MB PPT) [file pone.0009305.s004.ppt]

## Slide 1
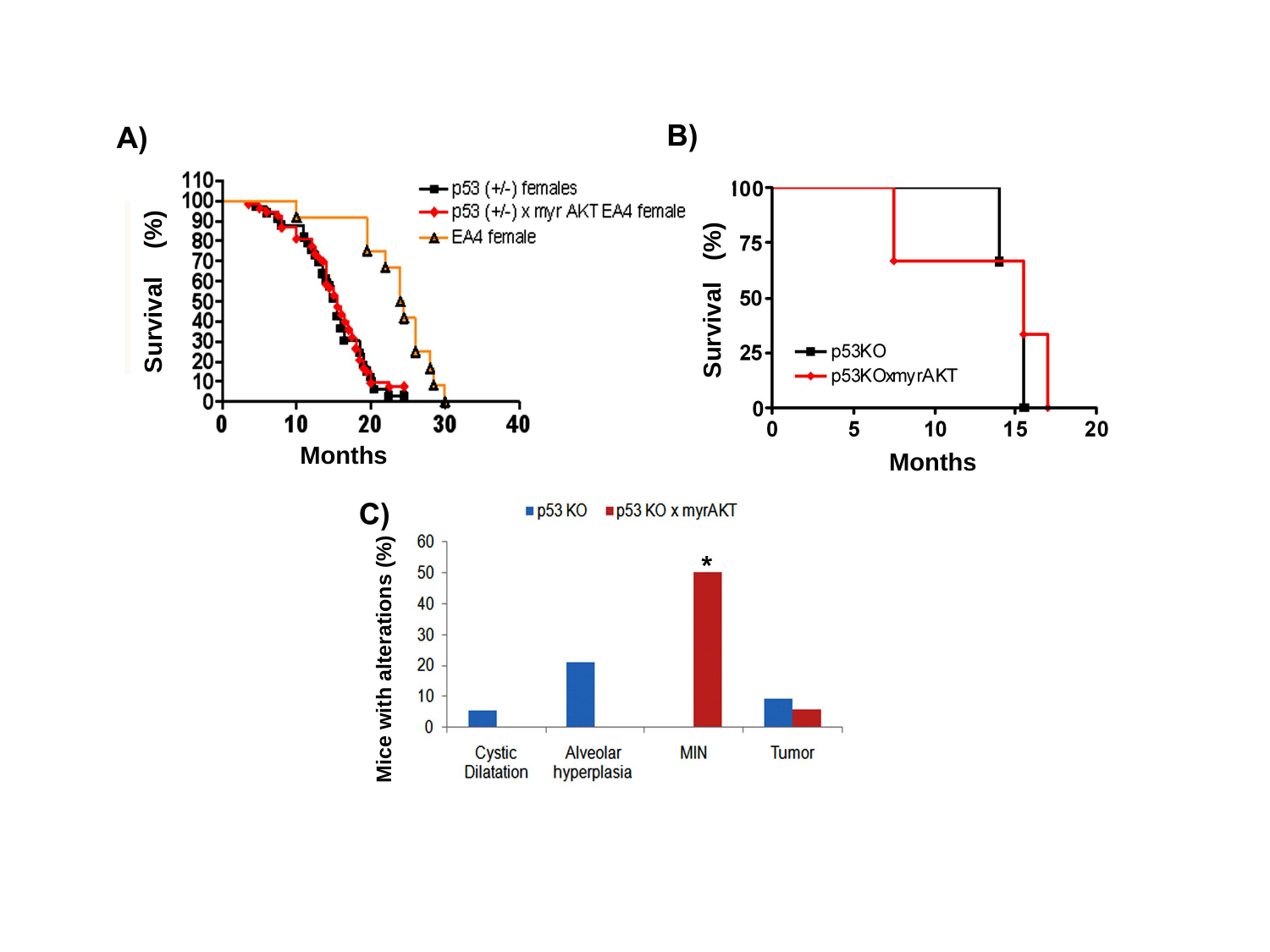

Survival (%)
 Survival (%)
 Months
 Months
Mice with alterations (%)

Supplement: Figure S5 — Survival and characterization of alteration in mammary gland of myrAKT transgenic mice in a heterozygous p53 (+/−) background. A) Tumor-free survival curves of p53(+/−), myrAKT and the double transgenic p53(+/−);myrAKT transgenic mice. Survival curves were computed using the Kaplan-Meier product-limit method. The numbers of mice are the following: p53(+/−) (n = 32), p53(+/−);myr-AKT(n = 49) and myrAKT (n = 12). B) Survival of mice that have developed mammary gland tumors. The numbers of mice are the following: p53(+/−)(n = 3); p53(+/−);myrAKT (n = 3). C) Incidence of preneoplastic and neoplastic lesions in p53(+/−) and p53(+/−);myr-AKT transgenic mice. (0.61 MB PPT) [file pone.0009305.s005.ppt]

## Slide 1
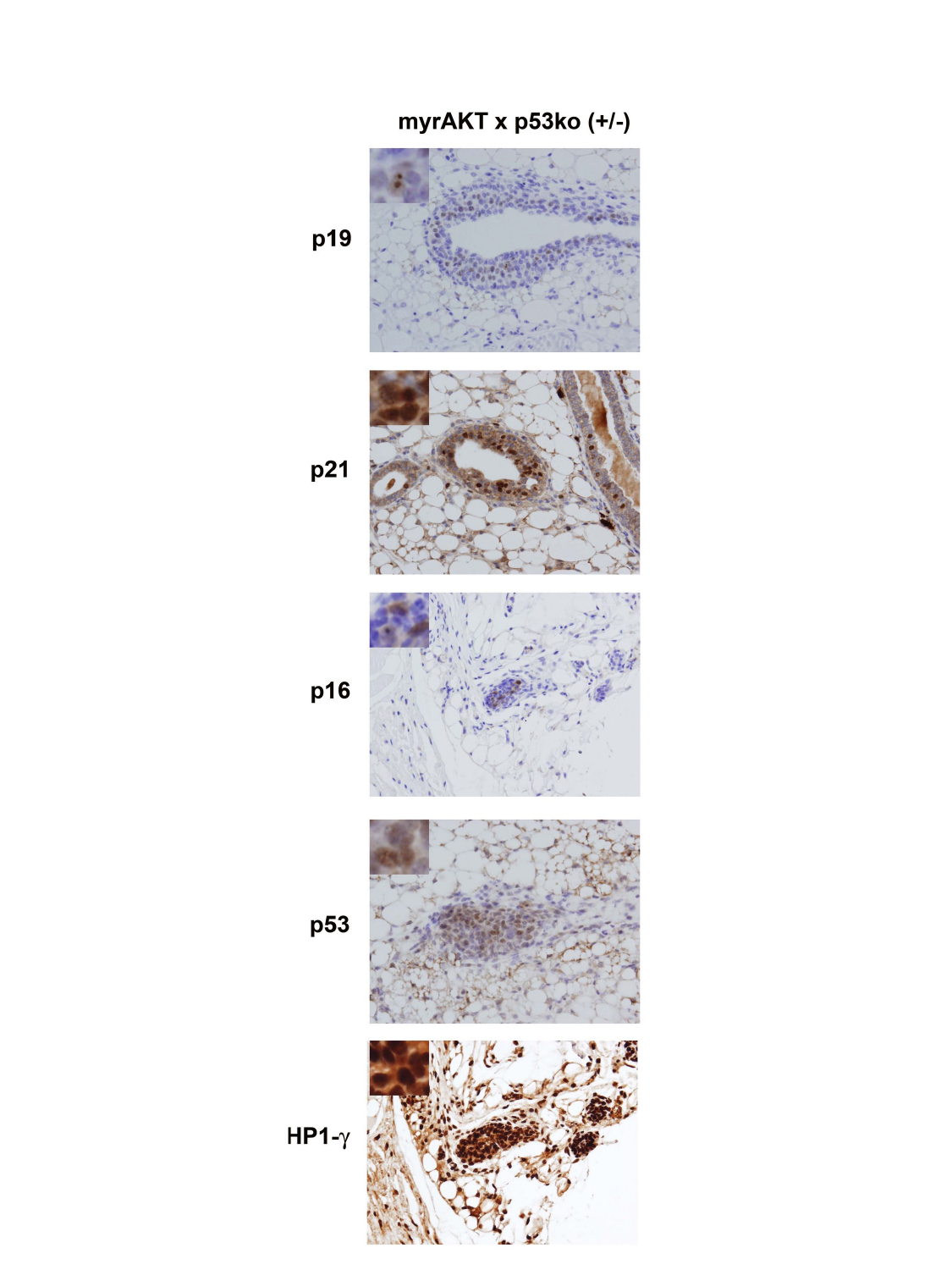

Supplement: Figure S6 — Expression of senescence markers in MIN from myrAKT in wild type, p53(+/−) or p53R172H background. Immunohistochemical staining of MIN with p19, p21 and p53. A 200× magnification was used and 400× magnification was inserted in the corner. (0.74 MB PPT) [file pone.0009305.s006.ppt]

## Slide 1
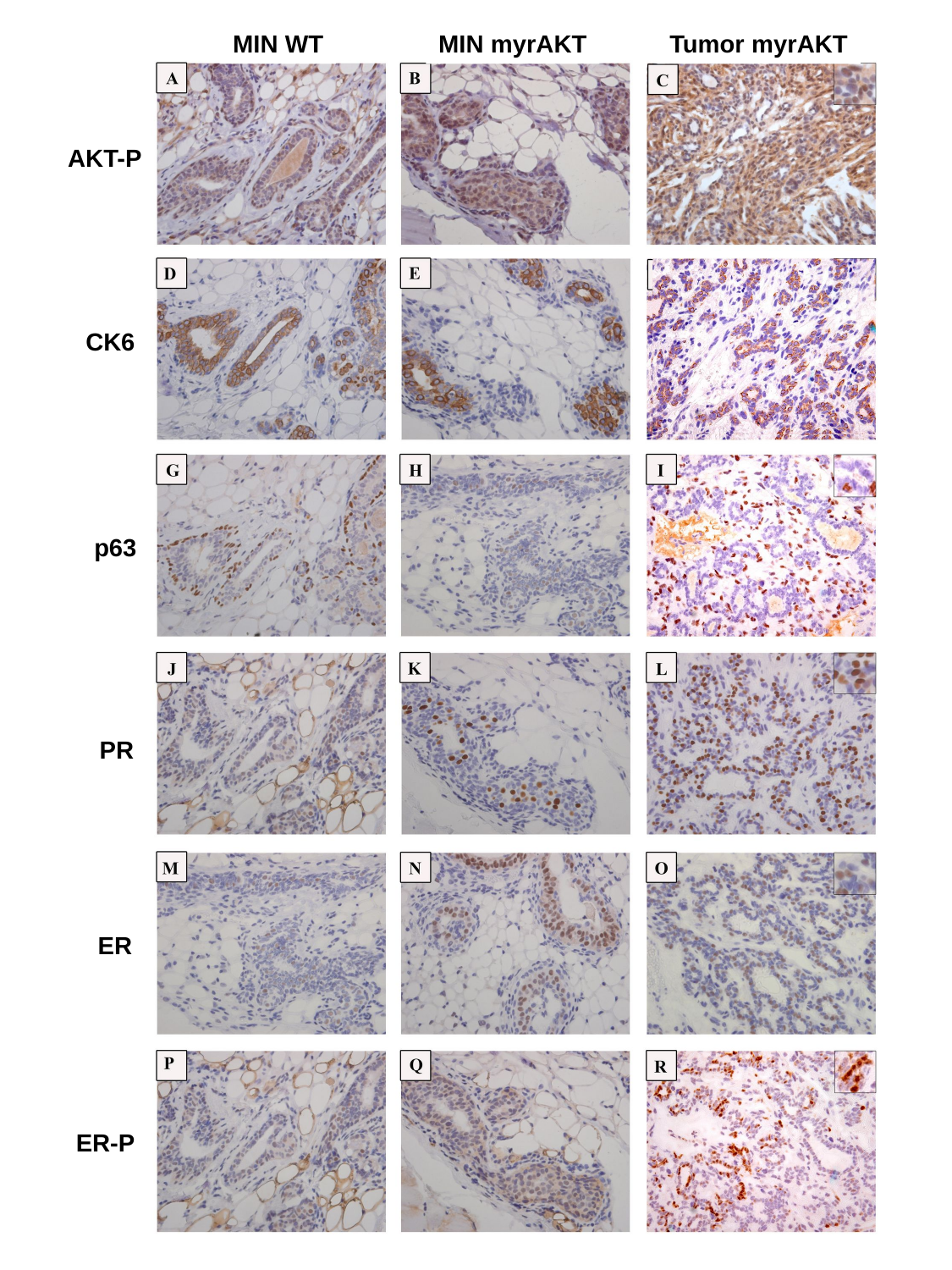

MIN WT
MIN myrAKT
Tumor myrAKT
AKT-P
CK6
p63
PR
ER
ER-P

## Slide 2
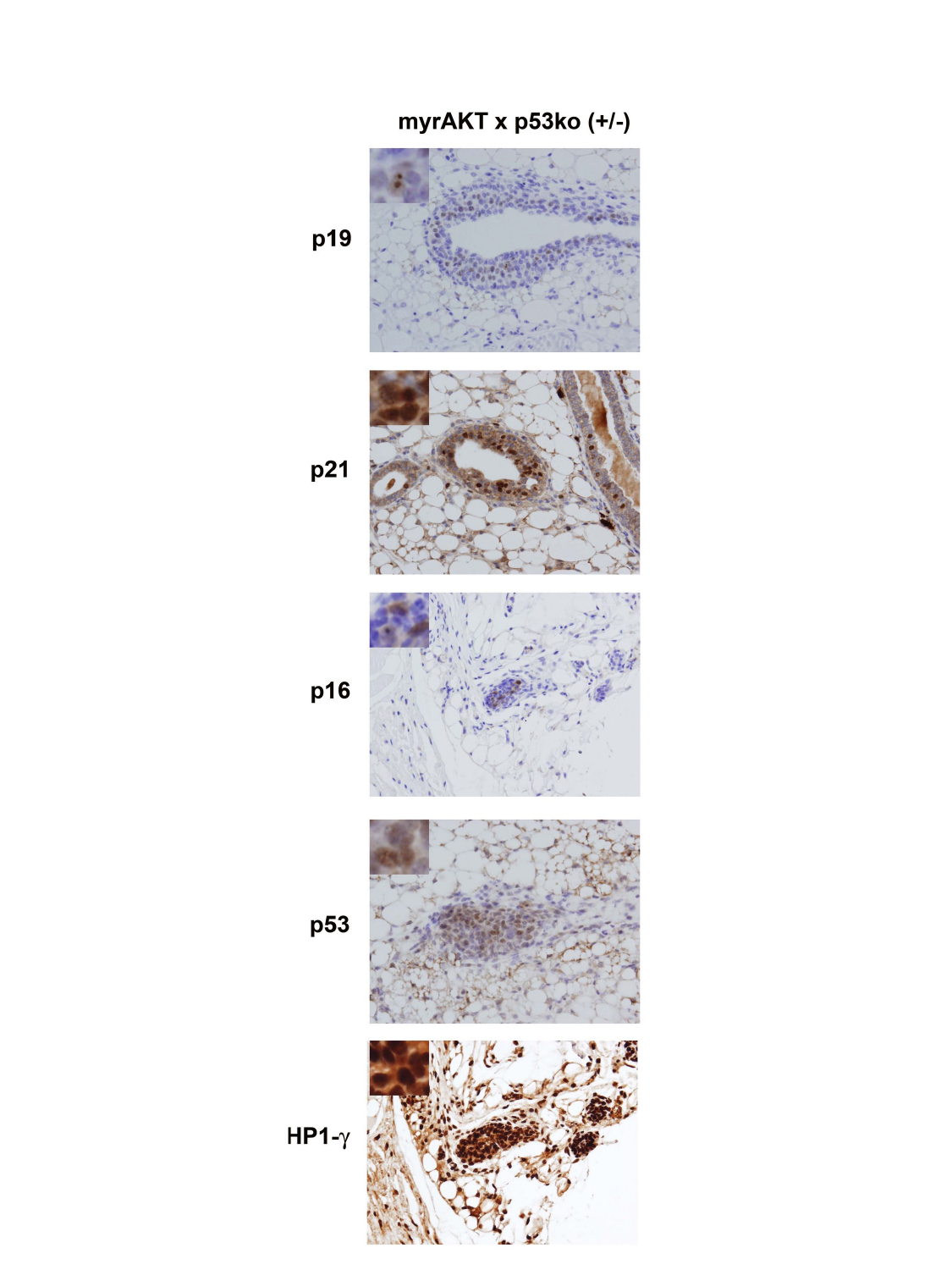

## Slide 3
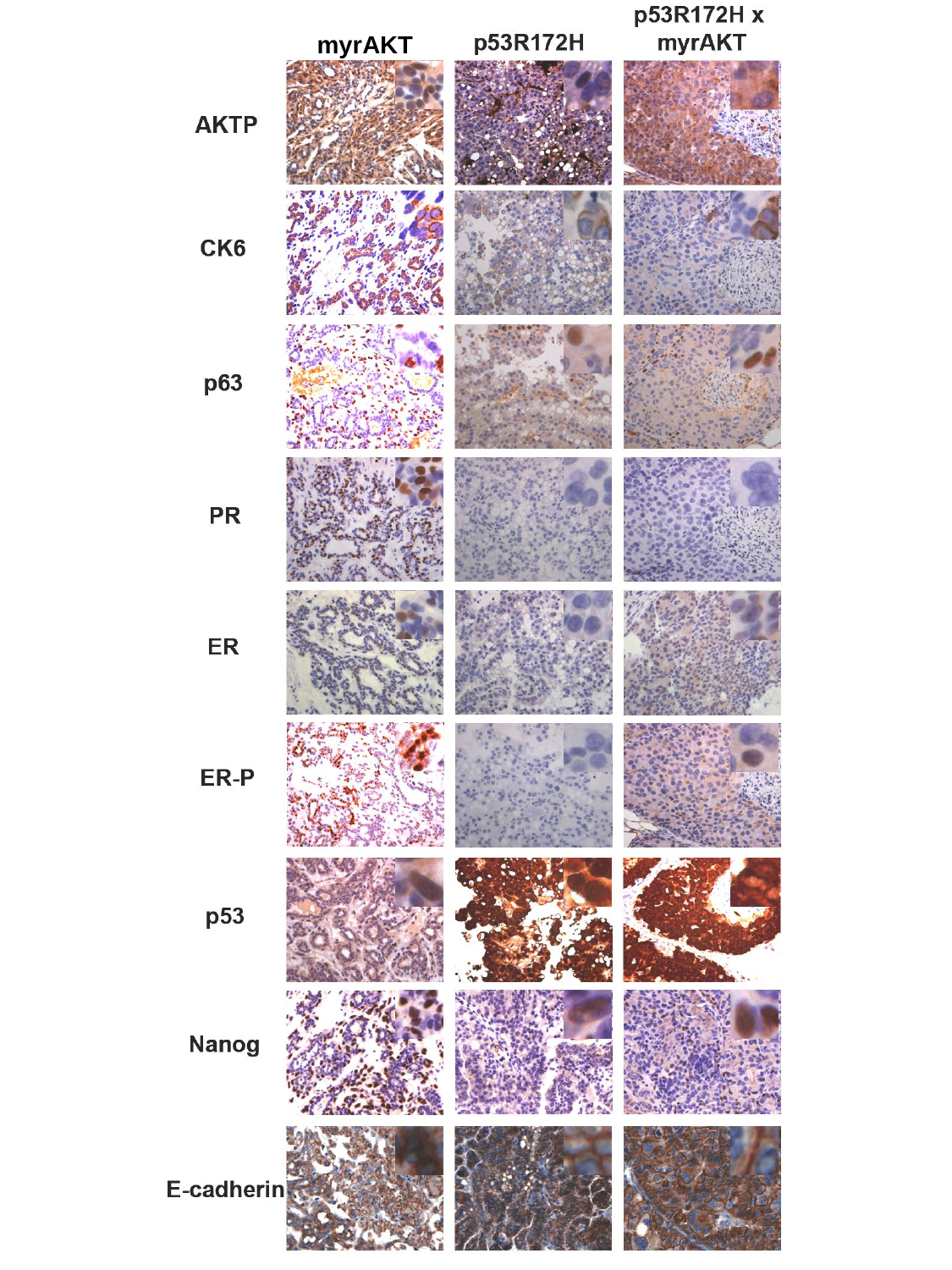

myrAKT

Supplement: Figure S7 — Molecular characterization of carcinomas in myrAKT, p53R172H and myrAKT;p53R172H transgenic mice. Immunohistochemical staining with AKT Ser473, cytokerin 6, p63, progesterone receptor (PR), estrogen receptor alpha (ER), phosphoS167ER, p53, nanog and E-Cadherin-. A 200× magnification was used and 400× magnification was inserted in the corner. (8.57 MB PPT) [file pone.0009305.s007.ppt]
